# Myocardial Functional Imaging in Pediatric Nuclear Cardiology

**DOI:** 10.3390/jcdd10090361

**Published:** 2023-08-24

**Authors:** Marco Alfonso Perrone, Andrea Cimini, Maria Ricci, Milena Pizzoferro, Maria Carmen Garganese, Massimiliano Raponi, Orazio Schillaci

**Affiliations:** 1 Department of Pediatric Cardiology and Cardiac Surgery, Bambino Gesù Children’s Hospital IRCCS, 00165 Rome, Italy; 2 Division of Cardiology and CardioLab, Department of Clinical Sciences and Translational Medicine, University of Rome Tor Vergata, 00133 Rome, Italy; 3 Nuclear Medicine Unit, St. Salvatore Hospital, 67100 L’Aquila, Italy; 4 Nuclear Medicine Unit, Cardarelli Hospital, 86100 Campobasso, Italy; 5 Division of Nuclear Medicine, Bambino Gesù Children’s Hospital IRCCS, 00165 Rome, Italy; 6 Health Directorate, Bambino Gesù Children’s Hospital IRCCS, 00165 Rome, Italy; 7 Department of Biomedicine and Prevention, University of Rome Tor Vergata, 00133 Rome, Italy

**Keywords:** nuclear imaging, pediatric cardiology, scintigraphy, positron emission tomography, congenital heart disease

## Abstract

The role of nuclear medicine in pediatric cardiology has grown rapidly over the years, providing useful functional and prognostic information and playing a complementary role to morphological imaging in the evaluation of myocardial perfusion, cardiovascular inflammation and infections, and cardiac sympathetic innervation. The aim of this narrative review is to summarize and highlight the most important evidence on pediatric nuclear cardiology, describing clinical applications and the possibilities, advantages, and limitations of nuclear medicine techniques. Moreover, a special focus will be given to the minimization of radiation exposure in pediatric nuclear cardiology imaging, a critical topic in children.

## 1. Introduction

Significant improvements in the clinical and surgical management of pediatric patients with cardiological disease (congenital and acquired) have led to improved survival. Advanced noninvasive cardiac imaging has played a pivotal role in this complex scenario, supporting preoperative decision-making and life-long surveillance in an increasing number of surviving patients. Multimodality cardiac imaging significantly contributes to individualized diagnosis and management for each patient, allowing a tailored treatment with consequent improved prognosis and clinical outcomes. In this context, echocardiography, computed tomography (CT), and magnetic resonance imaging (MRI) are very important imaging diagnostic techniques [1] in pediatric patients, but nevertheless, sometimes, they have important drawbacks. The echocardiogram is extremely operator-dependent, failing in cases of poor echo windows [2,3]. High heart rates represent one of the most important limitations of CT (this imaging technique also has high radiation doses of up to 13 mSv) [2,3]. For magnetic resonance, pacemakers and other implanted devices, if not compatible, are well-known contraindications, and furthermore, the execution of the exam can take a long time [2,3,4]. Advanced imaging techniques could be considered key elements for allowing the practice of “precision medicine” before, during, and after therapy. Among cardiac noninvasive imaging techniques, nuclear medicine provides useful functional and prognostic information complementary to that obtained using anatomic imaging and is useful in cases of limitations of other imaging techniques [3,4,5]. Nuclear imaging is playing a growing role in the evaluation of cardiac disease in the pediatric population, especially with the technological evolution of detectors and hybrid machines, which have a reduced amount of radiation exposure and improved diagnostic accuracy [4,5,6]. In adults, the main clinical applications include myocardial perfusion imaging, myocardial viability, cardiovascular infection and inflammation evaluation, and cardiac sympathetic innervation assessment. In the pediatric field, radiation exposure is a critical issue, but new-generation scanners use significantly decreased radiation doses, and the therapeutic clinical benefits overcome potential radiation risks considering the complex patient condition. The aim of this narrative review is to summarize and highlight the most important evidence on pediatric nuclear cardiology, describing clinical applications and the possibilities, advantages, and limitations of nuclear medicine techniques.

## 2. Myocardial Perfusion Imaging (MPI) in Pediatric Nuclear Cardiology

### 2.1. Indications

According to several studies, the main indications of myocardial perfusion imaging (MPI) in pediatric nuclear cardiology are:

- Kawasaki Disease (KD): MPI with Single Photon Emission Computed Tomography (SPECT) is used to detect myocardial ischemia and/or myocardial infarction in pediatric patients with KD. In fact, coronary arteries involvement frequently occurs without prompt treatment, with the development of coronary aneurysms in up to 25% of untreated children. Spontaneous regression of coronary abnormalities is observed in approximately two-thirds of patients during the first year after the acute illness, but long-term coronary stenosis may develop in some patients, even after aneurysm regression. Nuclear imaging is used particularly in patient follow-up. For example, [^18^F]Fluorodeoxyglucose (FDG) Positron Emission Tomography (PET) can also be used in these children for the assessment of myocardium viability [3,6,7,8].
- Congenital anomalies of the coronary arteries: These findings are sometimes reported in echocardiographic reports. For example, anomalous aortic origin of a coronary artery from the opposite sinus of Valsalva (AAOCA), associated with intramural or interarterial course, is increasingly diagnosed incidentally in children, but the related clinical risks are not well defined [3,6,9,10]. A fundamental exam in the assessment of these patients includes the evaluation of regional myocardial perfusion at rest and during physical or pharmacological stress, such as SPECT. The use of MPI has been reported in other rare congenital coronary diseases such as anomalous origin of the left coronary artery from the pulmonary artery (ALCAPA), Williams syndrome with coronary involvement, myocardial bridging, and complex CHD such as pulmonary atresia-intact ventricular septum with right ventricular coronary sinusoids or tetralogy of Fallot with coronary anomalies [3,10].

In particular, in anomalous origin of the left coronary artery from the pulmonary artery (ALCAPA), MPI with SPECT is often utilized in the post-operative follow-up in order to assess the extension of the ischemic myocardium area. Moreover, it is helpful for the preoperative evaluation of myocardium viability; patients showing radiopharmaceutical uptake of 50% or more in the left ventricle could benefit from any revascularization procedure [11,12]. [^18^F]FDG PET is also used in children to assess myocardium viability [12]. In fact, in these patients, identifying hibernating myocardium is useful for evaluating the chances of recovery after surgical repair. This can be detected by nuclear imaging as a mismatch between reduced rest perfusion and enhanced glucose uptake on PET imaging [3,12].

Transposition of Great Arteries (TGA): In the arterial switch operation for TGA, the coronary arteries are reimplanted at the time of surgery and may be prone to abnormal vasodilation, kinking, or failure to grow at the anastomosis level. Complications arising early after surgery often lead to severe hypoperfusion, fixed or reversible, and a poorer prognosis [2,13]. Several studies have demonstrated that MPI with SPECT or PET is very useful in these patients [2,4,13].

- Cardiomyopathies: For the assessment of the extent of myocardial damage in pediatric patients with cardiomyopathies, myocardial perfusion SPECT offers important information regarding cardiac pump function and myocardial function [6,14]. In particular, in hypertrophic cardiomyopathy (HCM), myocardial ischemia has been suggested to contribute to the pathophysiology of the disease and appears to be related to decreased subendocardial perfusion in the hypertrophied segments, compression of intramural small vessels, and myocardial bridging. MPI can contribute as a reliable noninvasive method for the detection of myocardial ischemia, adding to risk stratification and treatment [14,15].
- Heart transplantation: One of the long-term complications of heart transplantation is the development of cardiac allograft vasculopathy. In children, this complication is a major cause of death and retransplantation. The disease involves both distal and proximal coronary arteries and is associated with functional anomalies such as systolic dysfunction and increased filling pressures. Alongside cardiac catheterization, which is the recommended method, but is invasive and uses radiation, nuclear imaging can be used in follow-up [3,16,17].

### 2.2. SPECT Radiopharmaceuticals

For MPI with SPECT, the most used radiopharmaceuticals are technetium-99m (^99m^Tc) labeled radiopharmaceuticals (Sestamibi and Tetrofosmin) and, less often, thallium-201 (^201^Tl) chloride.

^99m^Tc-2-methoxyisobutylisonitrile (Sestamibi) and ^99m^Tc-1,2-bis[bis(2-ethoxyethyl) phosphino] ethane (Tetrofosmin) are cationic complexes that diffuse passively through the capillaries and cell membranes; subsequently, Sestamibi is retained in mitochondria, and Tetrofosmin localizes within the cytosol instead. Myocardial uptake of Sestamibi and Tetrofosmin augments with an increase in perfusion and reflects myocardium viability [18,19].

^201^Tl is a potassium analogue and consequently, its uptake in myocytes is mediated by sodium/potassium adenosine triphosphate (ATP) transporter. In nuclear cardiology, ^201^Tl chloride is utilized to evaluate myocardial viability and ischemia for its differential washout between regions of high and low blood flow (redistribution) [18,20].

Regarding MPI with SPECT in pediatric patients, ^99m^Tc labeled radiopharmaceuticals have better characteristics in comparison to ^201^Tl chloride. The main drawbacks of ^201^Tl are a higher physical half-life (^201^Tl half-life is about 73 h compared to ^99m^Tc half-life, which is about 6 h) and a higher radiation burden for children. Moreover, ^99m^Tc presents a better gamma energy emission (140 keV), which is more appropriate for providing Gated-SPECT analysis and evaluating the small hearts of pediatric patients.

**Figure 1:**
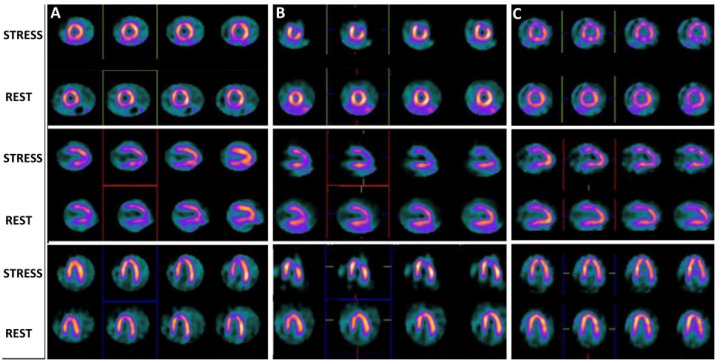
(**A**): Normal pattern of left ventricle stress and rest myocardial perfusion in a patient affected by Kawasaki disease. (**B**): Reversible myocardial perfusion defect (as for ischemia) in the antero-lateral wall of the left ventricle in a patient with Kawasaki disease. (**C**): Stress/rest myocardial perfusion scintigraphy showed mild multiple regional abnormalities of myocardial perfusion in a heart transplant patient.

Typically, perfusion imaging requires a stress phase and a rest phase. SPECT with ^99m^Tc labeled radio compounds needs two injections of ^99m^Tc-Sestamibi or ^99m^Tc-Tetrofosmin, one for each phase (Figure 1). Instead, SPECT with ^201^Tl chloride requires only one injection of the radiopharmaceutical due to its redistribution.

Regarding the injected dose, the ALARA (“as low as reasonably achievable”) principle must be followed in order to minimize radiation exposure. In general, a dose reduction could be performed with algorithms (for example, “EANM dose calculator”) or with the EANM Dosage Card [5,21,22].

### 2.3. Patient Preparation

When stress testing is scheduled, fasting is usually required if sedation is planned, according to anesthesiological indications [1]. In this context, motionless acquisition is one of the most important concerns during MPI in pediatric nuclear cardiology. Sedation is useful, especially for younger children (up to 5–6 years of age). Furthermore, in the case of neonates or infants, it is possible to obtain a good acquisition of images by taking advantage of post-feeding sleep [1]. Whenever possible, the interruption of beta-blockers (and other drugs that may interfere with heart rate) is required [1].

### 2.4. Stress Testing: Special Focus on Adenosine and Regadenoson

Stress testing for MPI in nuclear cardiology could be performed with the following:

- Physical stressor (preferably, for example, cycle ergometer or treadmill);
- Pharmacological stressor (typically dipyridamole, adenosine, or dobutamine).

Although physical stress gives more physiological information, it requires high compliance from patients. In this context, in pediatric patients, it may be arduous to reach the maximum predicted heart rate required to highlight abnormalities of coronary flow reserve [5]. Therefore, MPI should be performed by well-trained staff with high pediatric expertise to obtain the maximum cooperation of the young patients, limiting pharmacological stress in those children who are not able to perform an adequate physical stress test.

Adenosine is the most used pharmacological stressor in pediatric patients. This nucleoside has direct actions on A2a receptors (causing coronary vasodilatation) and a short half-life in the bloodstream (about 10 s); hence, side effects (correlated with the activation of A1AR, A2B, and A3AR, comprise flushing, headache, chest discomfort, dyspnea, gastrointestinal discomfort, lightheadedness/dizziness, AV block, paresthesia, hypotension, and arrhythmias) are usually mild and quickly disappear [5,23]. Adenosine is infused intravenously (0.14 mg/Kg/min over 6 min). The main contraindications are a history of reactive airway disease, second- or third-degree AV block, acute coronary syndromes, systolic BP < 90 mmHg, especially in case of autonomic dysfunction, hypovolemia, left main coronary stenosis, stenotic valvular heart disease, pericarditis, and cerebrovascular insufficiency. Xanthine interferes with adenosine; therefore, medication or foods/beverages containing xanthine should be avoided [23,24,25].

To the best of our knowledge, there is no report on the utilization of regadenoson as a pharmacological stressor for MPI in pediatric nuclear cardiology. Nevertheless, its favorable features may lead us to suggest the utilization of regadenoson in children in these exams. Regadenoson is a highly selective A2a receptor agonist, with a weaker affinity for A1 receptors and negligible binding to A2b receptors; hence, regadenoson has more favorable side effects in comparison to adenosine (in particular, AV block is less frequent with regadenoson) [23,26]. Moreover, there are studies demonstrating its feasibility in pediatric patients concerning its utilization as a pharmacological stressor in myocardial stress perfusion magnetic resonance [27,28,29,30].

### 2.5. SPECT and Technical Considerations on Image Acquisition and Processing

In general, SPECT acquisition is performed with a two-head gamma camera with an L configuration using a 180-degree rotation from the right anterior oblique to the left posterior oblique. In order to optimize SPECT imaging with an improved spatial resolution (and consequently improving the diagnostic accuracy of lesions detection in small hearts), the resolution could be improved using iterative algorithms in imaging reconstruction [31].

To date, attenuation correction (AC) of SPECT images is performed with algorithms using computed tomography (CT) or iterative reconstruction techniques [32]; nevertheless, AC with CT should be avoided in children in order to avoid additional radiation exposure [33].

For parameters deriving from Quantitative Gated SPECT (QGS), physicians should be aware of the possible overestimation of the left ventricular ejection fractions, measured by QGS software, which are often higher in small hearts [34]. Furthermore, Quantitative Perfusion SPECT (QPS) analysis is critical in children due to the absence of reference polar maps in pediatric nuclear cardiology.

It is important to underline that many pediatric patients could benefit from cardiac-centered gamma cameras, which are increasingly used in Europe. As a matter of fact, these gamma cameras have favorable features for MPI SPECT in children, such as increased spatial resolution in comparison to conventional gamma cameras and enhanced myocardial counts sensitivity that allows a reduced dose of the radiopharmaceuticals injected (and consequently a reduction in radiation exposure) [35].

### 2.6. PET Radiopharmaceuticals

MPI with PET has an established role in adults due to its favorable features, such as a higher spatial resolution in comparison to SPECT and its ability to provide accurate quantitative data of myocardial blood flow [20,36]; however, there are few studies regarding MPI with PET in pediatric patients.

The majority of these studies are focused on [^13^N] Ammonia ([^13^N]NH_3_), in particular in the evaluation of pediatric patients with coronary abnormalities [37] and after arterial switch operation for TGA. For example, Bengel et al., through the quantitative analysis of myocardial blood flow with [^13^N]NH_3_ PET, demonstrated a significant reduction of coronary reserve in a group of 22 asymptomatic children who underwent the arterial switch operation 10 ± 1 years before, in comparison to a control group composed of 10 healthy adults [38].

The synthesis of [^13^N]NH_3_ needs an on-site cyclotron due to the short half-life of ^13^N (9.96 min). The entire mechanism of localization in the cell of this radiopharmaceutical is not fully known; [^13^N]NH_3_ diffuses passively through the cell membrane, and then it undergoes trapping with the conversion of NH_3_ to glutamine, glutamic acid, and carbamoyl phosphate [39,40,41,42,43].

^82^Rubidium (^82^Rb), a PET tracer used in MPI, is a potassium analog, and its cellular uptake depends on sodium/potassium ATP transporter. ^82^Rb has a short half-life (1.16 min), which is the reason why its generator can be utilized only with the CARDIOGEN-82 ^®^ infusion system. Moreover, the ^82^Rb half-life allows a short duration of the exam (35–45 min for both rest and pharmacological stressor phases) [44]. Regarding the application of ^82^Rb PET in children, only one study has been performed [45] with promising results concerning the detection of coronary artery disease, demonstrating feasibility in pediatric patients (Table 1).

## 3. Other Applications in Pediatric Nuclear Cardiology

### 3.1. Myocardial Viability

The evaluation of myocardial viability has an important role in determining the efficacy of coronary surgery in pediatric patients with Kawasaki disease, ALCAPA, and TGA. SPECT with ^201^Tl could be performed [1,2,46,47,48] for this purpose, but to date, [^18^F]FDG PET is the gold standard for the assessment of myocardial viability due to its advantages, such as better spatial resolution and faster exam protocol. [^18^F]FDG is an analogue of glucose, and its cellular uptake is mediated by glucose transporters (principally GLUT 1 and 4), thus reflecting the cardiac myocytes’ vitality [49,50]. Synthesis of ^18^F depends on cyclotron, and its half-life is 110 min.

### 3.2. Infections and Inflammations

**Figure 2:**
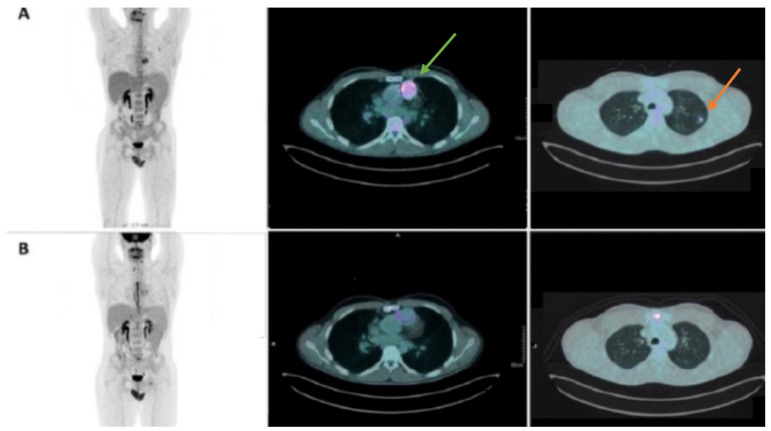
(**A**) (MIP and fused axial [^18^F]FDG PET-TC images): [^18^F]FDG PET images show increased radiopharmaceutical uptake around the aortic valve prosthesis (revealing an infective process, positive for Granulicatella adiacens; green arrow) associated with a lung septic embolism (orange arrow). An adequate preparation before PET imaging (consisting of a very low carbohydrate, high protein, and high-fat diet the day before, followed by fasting overnight on the day before imaging) allows for minimizing physiological 18F-FDG myocardial uptake. (**B**) (MIP and fused axial [^18^F]FDG PET-TC images): [^18^F]FDG PET images show no pathological uptake after surgical stent removal (as after surgical procedure) and medical treatment (with complete clinical response for lung embolism).

Valvular endocarditis is a possible complication of surgery or catheterization in pediatric patients with congenital heart disease [51]. The role of [^18^F]FDG PET combined with CT (PET/CT) in the diagnosis of endocarditis is well known [52]. Hypermetabolism of endocarditis may be detected by [^18^F]FDG PET due to the ability of cells involved in infections/inflammations to express high levels of GLUT transporters (especially neutrophils and monocities/macrophages) [53,54,55]. As regards its utilization in the pediatric population [^18^F]FDG PET/CT has shown promising results, detecting endocarditis and septic embolization foci with high sensitivity [51,56,57] (Figure 2).

PET/CT imaging with [^18^F]FDG is helpful in the assessment of large vessel vasculitis (such as Takayasu arteritis disease) in children [54,58,59]. In addition, this imaging technique may have a potential role in the assessment of a possible rejection after heart transplantation and in monitoring the evolution of rejection [17,60], detecting the inflammatory activity in the rejecting heart, with the most acute episodes occurring within the first 6 months after transplantation [60,61]. In this context, Sica et al. demonstrated the usefulness of [^18^F]FDG PET/CT for the early identification of post-heart transplantation lymphoproliferative diseases [62].

Somatostatin receptor imaging may be useful as well. Typically, rejection activity presents with lymphocyte infiltration, and these cells express somatostatin receptors. In the past, Mari Aparici et al. demonstrated the feasibility of scintigraphy with ^111^In-Octreotide (a somatostatin analogue) in order to evaluate lymphocytic activity during cardiac rejection [63]. The use of ^68^Ga-DOTA-peptides PET is conceivable for this purpose, with better hypothetical results. In comparison to scintigraphy, PET has a higher spatial resolution, and the affinity of ^68^Ga-DOTA-peptides in binding somatostatin receptors is higher than that of ^111^In-Octreotide [64].

### 3.3. Cardiac Sympathetic Innervation

**Figure 3:**
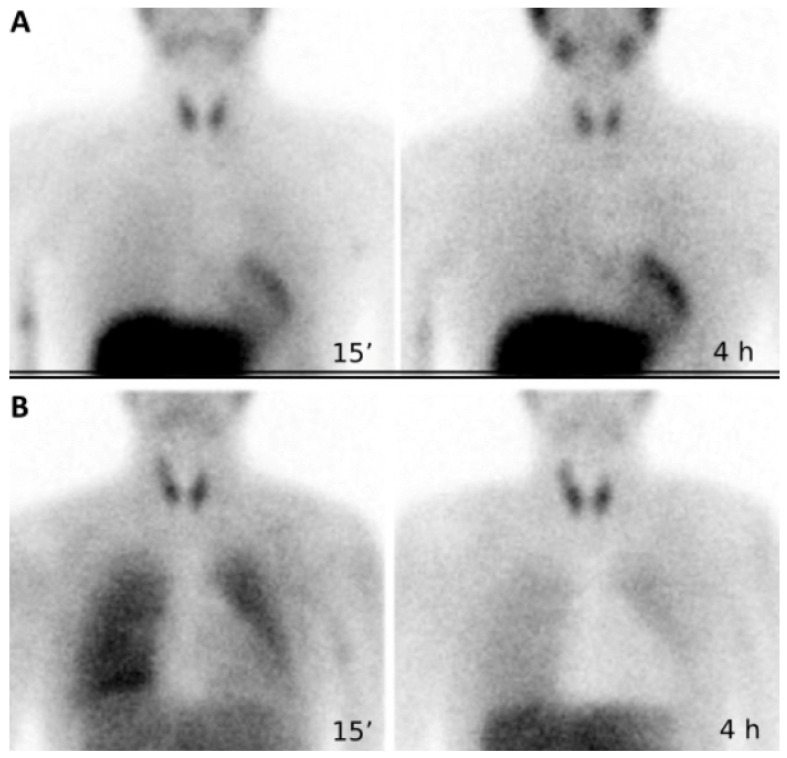
^123^I-mIBG scintigraphy is a useful method to assess cardiac reinnervation in pediatric patients undergoing heart transplantation, improving their clinical management. (**A**): Early and delayed images in the upper panel showed a significant myocardial ^123^I-mIBG uptake in a patient without chest pain who underwent heart transplantation for dilated cardiomyopathy. (**B**): No significant myocardial ^123^I-mIBG uptake is evident in images displayed in the lower panel. The patient underwent heart transplantation (9 years before) for univentricular heart, asymptomatic for chest pain, had a sudden cardiac death 8 months after ^123^I-mIBG scintigraphy.

Cardiac sympathetic innervation plays an important part in the regulation of myocardial blood flow [65]. Impairment of cardiac sympathetic nerve function may occur in pediatric patients:

- Subsequent to TOF or TGA correction and after heart transplantation, due to surgical damage [13,66,67] (Figure 3);

- With KD, probably due to coronary arteries stenosis [68].

Metaiodobenzylguanidine (MIBG) and meta-hydroxyephedrine (mHED) are both analogues of the neurotransmitter norepinephrine, their uptake in sympathetic neurons is mediated by the norepinephrine transporter. ^123^I-MIBG scintigraphy and [^11^C] mHED PET are used for the evaluation of cardiac sympathetic innervation. Their utilization in children for this purpose is well-documented [67,69,70].

### 3.4. Lung Scintigraphy

Lung scintigraphy with ^99m^Tc-macroaggregated albumin (^99m^Tc-MAA) plays an important role in lung perfusion studies. The particle size of MAA is 20–100 μm. After the tracer injection, 99mTc labeled particles are entrapped during their first transit through the pulmonary circulation in proportion to local blood flow. In children, dosing of ^99^mTc-MAA is weight-based to avoid a significant embolization in lung capillary vessels (more than 0.1% of total lung capillary vessels) [71] and radiation overexposure.

Branch pulmonary artery stenosis (BPAS) is a common post-operative complication in children with TOF. Lung scintigraphy with ^99m^Tc-MAA is commonly used to assess lung perfusion in pediatric patients with BPAS, playing a complementary diagnostic role with angiography (invasive and unsuitable for follow-up examinations) and transthoracic echocardiography (limited in some pediatric patients with obesity or post-operative changes such as fibrosis) [72,73]. Furthermore, the association with ventilation scintigraphy (performed with ^99m^Tc-Technegas) and SPECT may improve the diagnostic value of perfusion lung scintigraphy [74].

^99m^Tc-MAA scintigraphy may have a potential diagnostic role in the evaluation of children with primary pulmonary vein stenosis (PVS). PVS is a rare disorder caused by an intraluminal pulmonary vein obstruction caused by the neoproliferation of myofibroblasts. In a retrospective study, Drubach et al. demonstrated a significant correlation between findings in lung scintigraphy and angiography [75].

## 4. Conclusions

Nuclear medicine provides a wide range of solutions for diagnosing many pediatric cardiovascular diseases. As concerns imaging in pediatric nuclear cardiology, the best possible spatial resolution plays a fundamental role. Therefore, the development of new ^18^F-labeled radiopharmaceuticals for perfusion PET will improve diagnostic accuracy (^18^F has a shorter positron range in tissues in comparison to ^13^N and ^82^Rb and provides a higher spatial resolution imaging [76]). For this reason, [^18^F] Flurpiridaz and [^18^F] fluorobenzyltriphenyl-phosphonium ([^18^F]FBnTP) are promising tracers for MPI PET.

Hybrid system PET/magnetic resonance (MR) has a promising role, as well, with the combination of functional imaging (PET) and tissue characterization (MR); investigations are needed for application in pediatric cardiology.

## Figures and Tables

**Table 1:** Summary of advantages and drawbacks of MPI SPECT and PET radiopharmaceuticals.

Radiopharmaceutical	Advantages	Drawbacks
^99m^Tc-MIBI ^99m^Tc-Tetrofosmin	Availability in the majority of the nuclear medicine centers. ^99m^Tc has a better gamma energy emission in comparison to ^201^Tl.	The exam requires two injections. Poor spatial resolution: imaging needs to be optimized in order to improve spatial resolution.
^201^Tl chloride	The exam requires only one injection.	High physical half-life of ^201^Tl. High radiation burden for children. ^201^Tl has a suboptimal gamma energy emission.
[^13^N]NH_3_	PET with [^13^N]NH_3_ has a higher spatial resolution in comparison to SPECT.	Need for an on-site cyclotron. Reports on MPI in pediatric patients are limited.
^82^Rb	PET with ^82^Rb has a higher spatial resolution in comparison to SPECT. Short duration of the exam (35–45 min) due to its half-life of 1.16 min.	Need for its generator and CARDIOGEN-82 ^®^ infusion system. Reports on MPI in pediatric patients are very limited.
